# Factors influencing implementation of health-promoting interventions at workplaces: Protocol for a scoping review

**DOI:** 10.1371/journal.pone.0275887

**Published:** 2022-10-12

**Authors:** Kaung Suu Lwin, Aliza K. C. Bhandari, Phuong The Nguyen, Junko Saito, Akiko Yaguchi-Saito, Erika Ota, Taichi Shimazu

**Affiliations:** 1 Department of Global Health Policy, Graduate School of Medicine, The University of Tokyo, Tokyo, Japan; 2 Graduate School of Public Health, St. Luke’s International University, Tokyo, Japan; 3 Department of Health Policy, National Center for Child Health and Development, Tokyo, Japan; 4 Division of Surveillance and Policy Evaluation, National Cancer Center Institute for Cancer Control, Tokyo, Japan; 5 Division of Behavioral Sciences, National Cancer Center Institute for Cancer Control, Tokyo, Japan; 6 Faculty of Human Sciences, Tokiwa University, Ibaraki, Japan; 7 Department of Global Health Nursing, Graduate School of Nursing Science, St. Luke’s International University, Tokyo, Japan; 8 Tokyo Foundation for Policy Research, Tokyo, Japan; Manchester Metropolitan University - Aytoun Campus: Manchester Metropolitan University, UNITED KINGDOM

## Abstract

**Introduction:**

Health-promoting interventions at workplaces can be effective in modifying lifestyle-related behavioral risk factors for non-communicable diseases (NCDs). However, the interventions are not always successful in the real-world setting, and the evidence for effective strategies to implement the interventions has been limited. We propose a scoping review to identify the topics in need of study and areas for future research on barriers to and facilitators of the implementation of workplace health-promoting interventions.

**Materials and methods:**

This scoping review will explore these issues from the perspective of supply-side stakeholders, who have a direct role in the implementation of these interventions. An electronic systematic search of MEDLINE (using PubMed), Web of Science, and Scopus databases from 1986 to 2022, in accordance with the PRISMA-ScR guidelines, will be performed. Supplementary hand searching will be undertaken with reference lists from included articles and consulting with relevant stakeholders. Two authors will be responsible for individually screening the corresponding articles by first reading the titles and abstracts and then the full texts to assess whether they meet the inclusion criteria. Data extraction will be conducted using standardized data collection forms, and data analysis will be aligned to the consolidated framework for implementation research (CFIR), a determinant framework of factors affecting implementation, using a directed content analysis approach.

**Discussion:**

We will present the findings from this review at national and international conferences and submit them to a peer-reviewed journal for publication. Future workplace interventions will significantly benefit from this comprehensive scoping review to identify factors that enable improvement of the implementation, and the barriers to improvement, of evidence-based health-promoting interventions at workplaces.

## Introduction

Noncommunicable diseases (NCDs), including heart disease, stroke, cancer, chronic respiratory diseases, and diabetes, are the leading causes of mortality worldwide [1]. In 2015, approximately 40 million of 56 million global deaths were due to NCDs [2]. Although the most prevalent modifiable risk factors for NCDs are preventable by health-promoting interventions addressing diet, physical activity, weight control, tobacco use, and alcohol use [3], risky behaviors are still increasing worldwide. Globally, two-thirds of adults do not consume the recommended daily fruit and vegetable intake [4], one-third are physically inactive [5], one-third have a body mass index ≥ 25 [6], one-fourth of men are daily smokers [7], and 7.5% of adults have heavy episodic drinking [8].

Workplaces are valuable access points for providing health-promoting interventions for NCD prevention [9]. As many adult working populations spend most of their waking time at work [9], workplaces allow contact with a large number of the general population for a prolonged period, and the evidence of effective interventions to improve employees’ health has been accumulating [10–14]. However, these interventions are not always successful in real-world settings. For instance, a theory-based physical activity intervention at a worksite indicated no significant effect on metabolic equivalent minutes of activity [15]. This may have been due to the manner of delivery, as it was challenging for worksite managers to attract enthusiastic facilitators and encourage employees to participate in the program [15].

Workplace health promotion has expanded to address broader organizational and environmental factors, in addition to individual factors, since the Ottawa Charter for Health Promotion was adopted in 1986 [16]. Implementation Science focus on how to improve the ‘implementation’ of effective interventions, and enhancing the context in which the interventions are provided [17]. Strategies that improve the implementation of workplace health-promoting interventions can be effective when addressing these contexts including barriers to and facilitators of implementation [18]. However, a recent review found that evidence for effective strategies to promote the implementation of effective workplace-based health promotion interventions has been limited and inconsistent [19]. This may be because barriers and facilitators have not been comprehensively examined.

A previous systematic review identified multi-level factors at multiple phases of implementation but did not apply any implementation framework when summarizing the results [20]. The consolidated framework for implementation research (CFIR) is a conceptual framework developed to guide systematic evaluation of multilevel implementation contexts to identify factors that may influence the implementation and effectiveness of interventions [21]. The CFIR suggests that influences on implementation are complex, operating at multiple levels, and include individual, organizational, cultural, social, political, and other macro-level factors [21, 22]. Such conceptual frameworks not only increase research efficiency but also improve the generalizability and interpretability of research results [22]. Another systematic review focused on process evaluations for worksite health promotion programs using a theoretical framework and identified multi-level factors that influenced the implementation of health-promoting interventions at workplaces, including socio-political, organizational, operational, and individual factors [19]. However, they targeted only the studies that conducted the process evaluation and effect evaluation and did not include observational qualitative studies of the implementation of interventions in real-world settings. Therefore, this scoping review will collect and summarize a wide range of existing evidence regarding factors influencing the success and failure of workplace health promotion interventions using CFIR [21–23].

## Materials and methods

This scoping review will be conducted and reported according to the relevant sections of the Preferred Reporting Items for Systematic Reviews and Meta-Analyses Extension for Scoping Reviews (PRISMA-ScR) checklist (S1 Fig). Together with the PRISMA-ScR checklist, the Arksey and O’Malley framework will also be applied in this scoping review [24]. Based on the Arksey and O’Malley framework, there are six steps in this scoping review:

1. Identifying research questions
2. Identifying relevant studies
3. Study selection
4. Charting the data
5. Collating, summarizing, and reporting the results
6. Consultation

This scoping review is not registered in the International Prospective Register of Systematic Reviews (PROSPERO) as it does not register scoping reviews.

### Step (1): Identifying the research questions: Objectives

The objectives of this review are 1) to summarize the existing literature on barriers and facilitators influencing the implementation of health-promoting interventions at workplaces, and 2) to identify evidence gaps to make recommendations for future implementation research in health promotion at workplaces. We aim to answer the following research question: ‘What barriers and facilitators have influenced the implementation of health-promoting interventions at workplaces?’

### Step (2): Identifying relevant studies

In step (2), we will identify relevant studies based on the types of study, concept, participants, intervention(s), exposure(s), main outcome(s) and context.

#### Types of study to be included

The review will include all qualitative, quantitative, and mixed-method research studies evaluating barriers to, and facilitators of, implementation of workplace health-promoting interventions. Editorials, commentaries, and preliminary baseline studies performed before the implementation of the protocols will be excluded. Only articles available in English will be included in the study.

#### Concept

This study will focus on the barriers and facilitators influencing the implementation of health-promoting interventions at workplaces.

#### Participants/population

Participants will be supply-side stakeholders performing a direct role in the implementation of health-promoting interventions at workplaces, including, but not limited to, employers and management personnel at workplaces. Studies of only demand-side stakeholders (e.g., employees) will be excluded.

#### Intervention(s), exposure(s)

‘Interventions’ will be health-promoting interventions focusing on modifiable lifestyle-related NCD prevention to address diet, physical activity, weight control, and tobacco and alcohol use at workplaces (e.g., smoke-free policies at workplaces and workplace fitness programs).

Health-promoting interventions focusing only on injury-related and mental health-related prevention will be excluded.

#### Main outcome(s)

The main outcome will be any objective or subjective (self‐reported) measure of the results of implementation of health-promoting interventions targeting diet, physical activity, weight control, or tobacco or alcohol use at workplaces. Implementation outcomes will be categorized into eight constructs: acceptability, adoption, appropriateness, costs, feasibility, fidelity, penetration, and sustainability [25]. Studies will be excluded if they do not examine implementation outcomes; for example, studies that only evaluate subjects’ health outcomes. If implementation outcomes are reported as secondary outcomes, we will include the study and extract data only for implementation outcomes.

#### Context

Workplaces will include all employment sectors, such as manufacturing, health, education, business, information technology, retail, agriculture, construction, and mining. Studies will be excluded if they are conducted in communities or other settings rather than in workplaces.

### Step (3): Study selection: Searches

An electronic systematic search of PubMed, Web of Science and Scopus databases from 1986 to 2021 will be performed in accordance with the PRISMA guidelines. Workplace health promotion has expanded to address broader organizational and environmental factors, in addition to individual factors, since the Ottawa Charter was adopted in 1986 [16]. Thus, we will start the database searches from 1986 by considering multi-level factors, such as intervention characteristics, process, and organizational factors that extend beyond the individual, as one of the key issues in implementation science.

The review will include all research studies (quantitative, qualitative, and mixed methods studies) that address the following search items: 1) workplace, worksite, at work, and job site; 2) health-promoting intervention, wellness programs, physical activity, diet, weight control, tobacco, and alcohol use; 3) implementation outcome, acceptability, adoption, appropriateness, costs, feasibility, fidelity, penetration, and sustainability; and 4) factors, barriers, and facilitators. A complete detailed search strategy for each database is available upon request. Supplementary hand searching will be performed with reference lists from the included articles. We also intend to incorporate opportunities for exchanging the knowledge with stakeholders relevant to health-promoting interventions at workplaces [26]. These stakeholders will give us deeper insights into the results of this review. The two phases of study selection will involve a title/abstract screen and a full-text screen using open-access systematic review software [27]. Five review team members (KSL, AKC, PTN, JS, AY) will be responsible for individually screening the corresponding articles by first reading titles and abstracts, and then the full texts to assess whether they meet the inclusion criteria.

### Step (4) Charting the data

Data extraction will be carried out by review team members using standardized data collection forms (Table 1), and this will be cross-checked by another member. In cases of discrepancies in the extracted data consensus between the extracting authors, we will resolve disagreements by discussion and, where required. We will describe the reasons for exclusion in the PRISMA flow diagram. Charting the results can be an iterative process in which the collection forms will be continually updated [28].

**Table 1:** Data charting items for included articles.

Items	Sub-items
Study details	Author and date
	Country
	Study design
	Framework
	Study objective
	Study population
Intervention	What was implemented?
	Implementation setting (site, size)
	Implementation outcome reported by the author and categorized by specific terms (acceptability, adoption, appropriateness, costs, feasibility, fidelity, penetration and sustainability)
	Health outcomes reported by the author and whether they were worse, same, or better post-implementation.
Factors	Evaluation methods
	Barriers to implementation
	Facilitators of implementation

^a^Factors will be categorized according to the consolidated framework for implementation research with labeling barriers or facilitators.

Regarding the extraction of intervention items, we will extract information from the methods section of the article on the intervention components, intervention setting, implementation outcomes, and, if measured, health outcomes. In addition, when health outcomes are measured, we will also extract from the information in the results section of the article how the health outcomes changed after the intervention (whether they got worse, did not change, or improved after the implementation).

Regarding the extraction of factor items, barriers, and facilitators of implementation, we will conduct directed content analysis to identify and code influencing factors [29]. First, we will develop a codebook, using existing CFIR codes, that categorizes factors affecting implementation into five domains: the intervention characteristics, the organization’s outer and inner setting, the characteristics of the individuals involved, and the process of implementation [21]. We will also apply the International Standard Industrial Classification of All Economic Activities (ISIC) to classify the workplaces [30]. Next, we will read and highlight all text mainly from the Results section, but also in other sections when relevant, in each included study that represents influencing factors on implementation. Then, we code all the highlighted descriptions using the predetermined codes deductively. The five to ten studies will be pilot tested by two independent review team members, and inconsistencies will be resolved through discussion with updates to the codebook. Once consensus is reached, one of the review team members will independently code the studies, and another member will cross-check their work for validity and apply the final codes to each description. Descriptions that do not clearly fit into the predefined codebook will be coded as ‘other,’ and categorized into a new category named by the theme derived inductively from the descriptions.

### Step (5): Collating, summarizing, and reporting the results

Due to the anticipated inclusion of qualitative and quantitative studies within the scoping review, a narrative summary of results is most likely to be appropriate. We will provide a summary table for the barriers and facilitators identified in this review. In the table, a brief summary of descriptive data of each barrier and facilitator for predefined sub-constructs of the CFIR and newly developed code from individual studies will be illustrated. If appropriate, barriers and facilitators will be organized into multiple groups according to the frequency of reporting, and the health topic similarities and differences in implementation in workplaces will be highlighted. In addition, if appropriate, we will stratify the results by the three categories of health outcome change (worse, no change, better) and compare the barriers and facilitators of the articles on better health outcomes with those of the overall articles, and highlight their similarities and differences. If health outcomes worsened or did not change, we will also consider whether this was due to limited implementation, and if so, which barriers were the major contributors.

### Step (6): Consultation

Throughout our review process, we will consult with public health nurses who are engaging in support workplace to improve the health promotion implementation, and health managers engaging in actual health promotion interventions at workplace about their valuable insights.

### Ethics

Ethical approval for this study was not required, as this scoping review included only published data. Regarding consultation exercise, we aim to collect opinions rather than study data, and such an involvement activity does not require ethical approval.

## Discussion

This scoping review will identify any literature gaps and potential areas for future research on factors influencing the implementation of health-promoting interventions at workplaces. The strength of this scoping review is to comprehensively assess articles focusing on ‘implementation’ using the consolidated framework of implementation research by addressing multi-level factors such as intervention characteristics, process, and organizational factors that extend beyond the individual. The main limitation of this scoping review is that it will not assess the perspectives of demand-side stakeholders.

We will present the findings from this review at national and international conferences and submit them to a peer-reviewed journal for publication. Future workplace interventions will significantly benefit from this comprehensive scoping review to identify factors that will enable improvement of the implementation, and the barriers to improvement, of evidence-based health-promoting interventions at workplaces.

## Supporting information

S1 Fig PRISMA-ScR-P checklist. (DOCX)

S1 Table Database search strategy (PubMed). (DOCX)
