# Supplementary material for: Factors influencing implementation of health-promoting interventions at workplaces: Protocol for a scoping review
Source: PLoS One. 2022 Oct 12;17(10):e0275887. doi: 10.1371/journal.pone.0275887 (PMC9555663; doi:10.1371/journal.pone.0275887)
Supplement: S1 Table — (DOCX) [file pone.0275887.s002.docx]

**Supplementary (2): Database Search Strategy (PubMed)**

| **Search Strategy** | | |  |
| --- | --- | --- | --- |
| **S.N** | **Key component** | **Searched terms** |  |
|  |  |  |  |
| **1** | **Factors** | barrier* OR challeng* OR limit* OR complicat* OR obstacle* OR hindrance* OR hinder* OR obstruct* OR restrain* or restrict* OR interfer* OR influen* OR imped* OR facilitat* OR enable* OR benefit* OR promot* OR empower* OR accept* OR factor* OR determinant* |  |
|  |  |  |  |
|  |  |  |  |
|  |  |  |  |
|  |  |  |  |
|  |  |  |  |
|  |  |  |  |
|  |  |  |  |
|  |  |  |  |
| **2** | **Implementation** | “implementation science” OR implement* OR acceptab* OR satisfaction OR agreeability OR adoption OR uptake OR “knowledge translation” OR “intention to adapt” OR utilization OR appropriateness OR “perceived fit” OR relevance OR applicability OR compatibility OR fitness OR “cost-benefit analysis” or cost OR “cost-effect*” OR feasibility OR transferability OR practicability OR workability OR “actual fit” OR “actual utility” OR fidelity OR integrity OR “delivered as intended” OR “quality of program delivery” OR penetration OR “integration of practice” OR infiltration OR sustainability OR maintenance OR routinization OR durability OR institutionalization OR “capacity building” OR *continuation OR incorporation OR “sustained use” OR adhere OR participat* |  |
|  |  |  |  |
| **3** | **Health promotion interventions** | innovation OR EBP OR "evidence-based practice" OR “evidence based practice” OR "health promotion" OR "health management" OR "Health protection" OR “Well-being” OR obes* or "weight gain" or "weight loss" or overweight or "over-weight" or overeat* or "over eat" or "weight change*" or ((bmi or "body mass index") and (gain or loss or change)) or "primary prevention" or "preventive measure*" or "preventative measure*" or "preventive care" or "preventative care" or (obesity and (prevent* or treat*)) or exercise or "physical inactivity" or "physical activity" or "Motor Activity" or ("physical education and training") or "Physical Fitness" or sedentary or "Life Style" or "Leisure Activit*" or sport* or dancing or diet or nutrition* or "healthy eating" or fruit* or vegetable* or canteen or food or menu or calorie* or "energy intake" or "energy density" or eating or "feeding behavior" or "dietary intake" or "soft drink*"or soda or "sweetened drink*"or fat or confectionary or "feeding program*"or cafeteria* or ((smok* or tobacco or nictotine) and (cessation or stop* or quit* or abstin* or abstain* or reduc* or ex-smoker* or anti-smok*) or alcohol* or drink* or liquor* or beer* or wine* or spirit* or drunk* or intoxicat* or binge) |  |
|  |  |  |  |
| **4** | **Work place** | job OR workplace* OR worksite* OR work site* OR “work place*” OR “at work” OR “job site*” OR occupation* OR employ* |  |
